# Piezotransistive transduction of femtoscale displacement for photoacoustic spectroscopy

**DOI:** 10.1038/ncomms8885

**Published:** 2015-08-10

**Authors:** Abdul Talukdar, M. Faheem Khan, Dongkyu Lee, Seonghwan Kim, Thomas Thundat, Goutam Koley

**Affiliations:** 1 Department of Electrical Engineering, University of South Carolina, Columbia, South Carolina 29208, USA; 2 Department of Chemical and Materials Engineering, University of Alberta, Edmonton, Alberta T6G 2V4, Canada; 3 Department of Mechanical and Manufacturing Engineering, University of Calgary, Calgary, Alberta T2N 1N4, Canada; 4 Department of Electrical Engineering, Clemson University, Clemson, South Carolina 29634, USA

## Abstract

Measurement of femtoscale displacements in the ultrasonic frequency range is attractive for advanced material characterization and sensing, yet major challenges remain in their reliable transduction using non-optical modalities, which can dramatically reduce the size and complexity of the transducer assembly. Here we demonstrate femtoscale displacement transduction using an AlGaN/GaN heterojunction field effect transistor-integrated GaN microcantilever that utilizes piezoelectric polarization-induced changes in two-dimensional electron gas to transduce displacement with very high sensitivity. The piezotransistor demonstrated an ultra-high gauge factor of 8,700 while consuming an extremely low power of 1.36 nW, and transduced external excitation with a superior noise-limited resolution of 12.43 fm Hz^−1/2^ and an outstanding responsivity of 170 nV fm^−1^, which is comparable to the optical transduction limits. These extraordinary characteristics, which enabled unique detection of nanogram quantity of analytes using photoacoustic spectroscopy, can be readily exploited in realizing a multitude of novel sensing paradigms.

## Abstract (web summary)

Microelectromechanical systems—micrometre-sized devices with movable parts—make highly sensitive transducers. Here, the authors fabricate an integrated gallium nitride microcantilever and heterojunction field effect transistor that uses piezoelectric effects to measure displacement at the femtoscale level.

## Graphical abstract

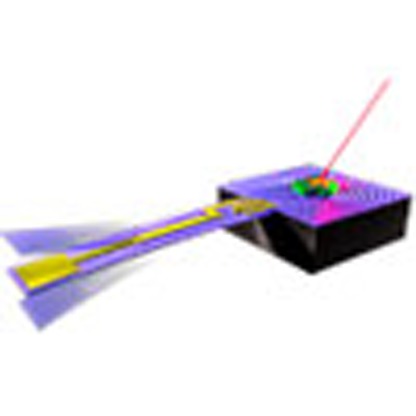

Development of ultrasensitive micro- and nano-electromechanical systems (MEMS/NEMS) has resulted in ultra-high detection sensitivity, offering sub nanometre scale displacement detection^1^,^2^,^3^, zeptogram level mass sensing^4^,^5^,^6^, single bio-molecular sensing^7^,^8^,^9^ and atomic resolution imaging^10^,^11^,^12^,^13^. Micro and nanocantilevers, as MEMS/NEMS transducers, have been used extensively for these sensing applications. Optical transduction of cantilever motion is almost exclusively used to achieve high deflection sensitivity (in the femtometre range), but it suffers from high power requirement, challenges with miniaturization and array-based operation^14^. Femtometre scale displacement detection using nanocantilevers operating at several hundred megahertz has been demonstrated^2^, but is limited by its challenging fabrication and integration schemes, coupled with complicacies of impedance matching for high frequency signal transmission. Si-based piezoresistive microcantilevers have been developed^15^,^16^,^17^, which are easily integrated for array-based operation, but have low sensitivity offering displacement resolution in the range of nanometres^18^. Instead of a simple piezoresistor, embedding a transistor at the base of the microcantilever (henceforth to be called a ‘piezotransistive' microcantilever) to transduce its deflection is an attractive way to dramatically improve its sensitivity by orders of magnitude^19^,^20^, since the gate can be utilized to control the charge carrier density and the mobility of the carriers in the channel.

Recently, metal oxide semiconductor field effect transistor (MOSFET) integrated Si cantilevers have been proposed with the goal of achieving very high deflection sensitivity while avoiding the challenges associated with the aforementioned techniques^17^,^19^. Although these microcantilevers showed high sensitivity in the nanometre range for step deflections, since its high sensitivity supposedly originated from trapping effects in the MOSFET it is difficult to reproduce these sensors, or operate them at high frequencies. Indeed, Si-based piezotransistive microcantilevers are theoretically incapable of exhibiting direct sensitivity enhancement through gate control, since the piezoresistive effects in Si originate from the variation in carrier mobility due to strain-related splitting of the conduction band minima energy levels^21^. On the other hand, piezotransistive cantilevers made of piezoelectric materials can directly utilize the charge density variation caused by the deflection-induced strain to exhibit high sensitivity with very high repeatability. Due to strong piezoelectric properties of AlN and GaN, AlGaN/GaN heterojunction^22^, provides a unique avenue to translate the static piezoelectric charge generated at the interface due to applied strain into a change in resistance of the two-dimensional electron gas (2DEG) formed at the interface^22^,^23^, since the generated piezoelectric charge can proportionately modulate the density of the 2DEG^20^,^22^,^23^. In addition to changing the carrier density, the applied strain can also change the carrier mobility by changing their effective mass^20^,^22^,^23^,^24^. The utility of AlGaN/GaN heterojunction-based piezoresistor (for step bending and dynamic deflection measurements) and piezotransistor (for static deflection measurements) has been demonstrated^20^,^24^,^25^, however, the effect of gate in enhancing displacement sensitivity down to femtometre range in high frequency dynamic deflection mode, with subsequent applications in unique analyte detection, has never been realized.

In the present work, we report on the ultra-high deflection sensitivity achieved using AlGaN/GaN heterojunction field effect transistor (HFET) embedded piezotransistive GaN microcantilever, which resulted in successful transduction of femtometre level displacement at the resonance frequency of the cantilever. The capability of measuring these extremely small displacements, verified independently through laser Doppler vibrometry studies, has enabled detection of nanogram-level explosives with high specificity using a novel surface-based photoacoustic spectroscopy^26^ technique.

## Results

### AlGaN/GaN HFET-embedded GaN microcantilever

**Figure 1:**
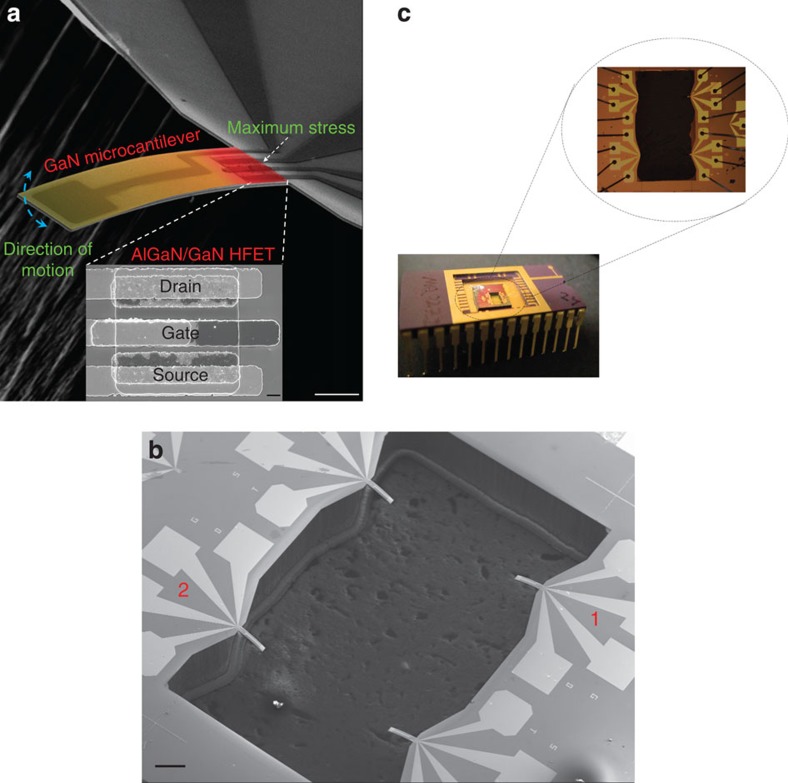
Images of AlGaN/GaN HFET-embedded GaN microcantilevers. (**a**) SEM image of GaN microcantilever with AlGaN/GaN HFET embedded at the base (scale bar, 50 μm). The inset shows a magnified section containing the HFET (scale bar, 2 μm). The microcantilever is coded with false colour along its length to show the stress distribution when it is deflected. (**b**) SEM image of a representative chip with four microcantilevers overhanging in a rectangular trench (scale bar, 500 μm). The microcantilevers investigated in this study are as marked 1 and 2. (**c**) Picture of a 28 pin DIP package with the microcantilever chip wire bonded. The inset shows a close-up of the wires bonded to the bias pads.

**Figure 2:**
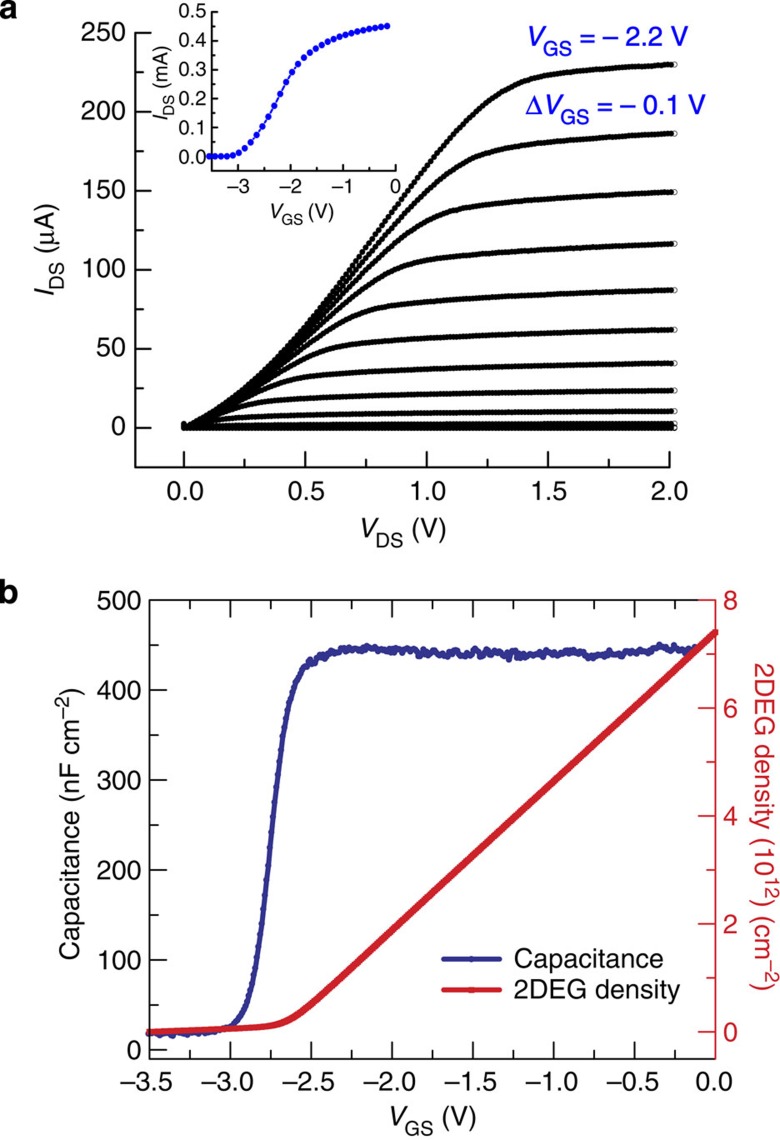
Electrical characteristics of AlGaN/GaN HFET. (**a**) Typical *I–V* characteristics of the AlGaN/GaN HFET deflection transducer for microcantilever 1. Inset shows the threshold voltage of −3.1 V for the device in the *I*_DS_–*V*_GS_ plot (measured for a *V*_DS_=0.5 V). (**b**) *C–V* profile of HFET transducer, which was processed to obtain the 2DEG density variation with gate bias (measured for a *V*_DS_=0.5 V).

Piezotransistive microcantilevers were fabricated using III-Nitride epitaxial layers grown on Si (111) substrate (bought from DOWA Semiconductor Akita Co., Ltd.). The overall layer structure consists of i-GaN (2 nm)/AlGaN (17.5 nm, 26% Al)/i-GaN (1 μm)/transition layer (1.1 μm)/Si (111) substrate (500 μm). The HFET was fabricated (in the Institute for Electronics and Nanotechnology (IEN), Georgia Institute of Technology) with initial 200 nm mesa etching followed by GaN cantilever pattern etched down using BCl_3_/Cl_2_-based inductively coupled plasma etch process. Ohmic contacts were formed with Ti (20 nm)/Al (100 nm)/Ti (45 nm)/Au (55 nm) metal stack deposition and rapid thermal annealing. Schottky gate contact was then formed with Ni (25 nm)/Au (375 nm) deposition. Finally, through wafer Si etch was performed by gas chopping etching process, also commonly known as Bosch process to release the microcantilevers (see Supplementary Fig. 1 for the detailed process flow). The fabricated microcantilevers had dimensions of 250 × 50 × 2 μm^3^, with the embedded HFET having a mesa dimension of 17 × 29 μm and a gate length of 5 μm. Figure 1a shows the scanning electron micrograph (SEM) of our fabricated self-sensing piezoelectric GaN microcantilever with the AlGaN/GaN HFET (bottom inset) fabricated at its base. The HFET is positioned to take advantage of the maximum stress occurring at the base due to microcantilever deflection, as shown by a colour map in Fig. 1, which is supported by COMSOL-based finite element simulations shown in Supplementary Fig. 2. Each chip has four similar microcantilevers (Fig. 1b), which are wire bonded to a 28 pin dual-in-line package (DIP) chip carrier as shown in Fig. 1c. The results presented in this article are from devices 1 and 2 as indicated in the SEM image of Fig. 1b. Typical *I*_DS_–*V*_DS_ and *I*_DS_–*V*_GS_ characteristics of the HFET (device 1), exhibiting good gate control, are shown in Fig. 2a. The low current level in *I*_DS_ is mostly due to smaller contact area and ohmic contact formation. Utilizing a negative gate bias (*V*_GS_), the piezoresistive effect is translated into a piezotransistive effect where the 2DEG carrier concentration (*n*_s_)^27^ is reduced, thus primarily increasing the Δ*n*_s_/*n*_s_ ratio (Δ*n*_s_ is the change in 2DEG concentration due to strain caused by deflection of the cantilever). Figure 2b shows the gate bias dependence of the 2DEG density, which was obtained for a particular gate bias by integrating the *C*–*V* characteristic. The capacitance was measured between the gate schottky contact and the source using an LCR meter (Model# HP4284A). The fractional change in resistivity of the intrinsic device *ρ*_int_ (due to external stress) is related to the 2DEG concentration *n*_int_ and the mobility *μ*_int_ as (detailed derivations are included in Supplementary Note 1):


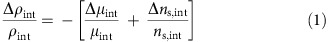


As *n*_s,int_ is controlled only by the gate voltage, and its change Δ*n*_s,int_, for a given gate voltage only depends on the change in piezoelectric-bound charge at the AlGaN/GaN interface due to stress (relevant equations are in ref. 24), the ratio Δ*n*_s_/*n*_s_ will increase as the gate voltage becomes more negative and *vice versa*. Since *μ*_int_ also decreases with reduced *n*_int_ (due to increased scattering)^27^, the ratio Δ*μ*_int_/*μ*_int_ will also increase with more negative gate voltage, causing Δ*ρ*_int_/*ρ*_int_ to increase strongly as *V*_GS_ becomes more negative. Clearly, to maximize Δ*ρ*_int_/*ρ*_int_, which ultimately controls the sensitivity, appropriate choice of *V*_GS_ is very important.

### Transduction of step deflection

**Figure 3:**
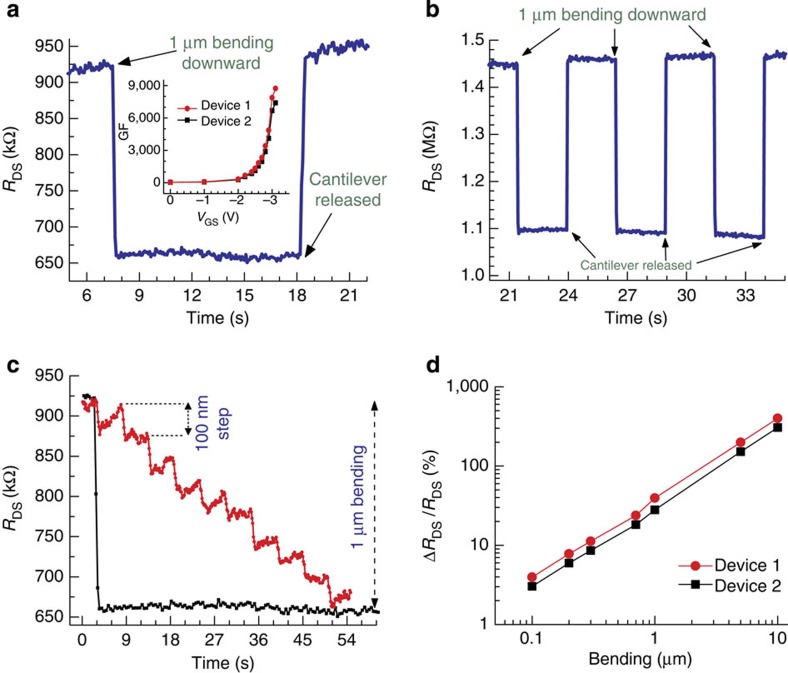
Nanoscale static deflection measurements (HFET readouts). (**a**) Step bending response for HFET 1 under applied biases *V*_DS_=30 mV and *V*_GS_=−3.0 V, when the tip of the cantilever was bent by 1 μm using the nanopositioner. Inset shows gate bias dependence of the gauge factor for both the HFET devices. (**b**) Multiple step bending responses of device 2 for 1-μm tip bending (*V*_DS_=30 mV and *V*_GS_=−3.0 V), showing measurement repeatability. (**c**) Response to cantilever bending in 10 steps of 100 nm each, showing repeatable and overall linear response demonstrating nanometre level deflection transduction with high sensitivity. (**d**) Plot of sensitivity (Δ*R*_DS_/*R*_DS_) versus microcantilever tip bending in the range 100 nm to 10 μm shows a linear response for both devices. *V*_DS_=30 mV and *V*_GS_=−3.0 V were maintained throughout the measurements.

The static deflection experiments were performed by controllably bending the free end of the microcantilever using a tungsten needle with a tip diameter of 12 μm. The needle was attached to a nanopositioner bought from Physic Instrument (Model# P-611 Z, PI Inc.) and controlled using Labview (experimental set-up is shown in Supplementary Fig. 3). A dual channel source measure unit (SMU) from Keithley (Model# 2612 A) was used to bias the HFET, and measure both the source–drain current and resistance. For device 1, under an applied gate bias of −3.0 V (close to the threshold voltage of −3.1 V) and a drain bias of 30 mV the drain–source resistance (*R*_DS_) of the HFET channel reduced by 39.3% when the tip of microcantilever was bent 1 μm downward, and recovered back to its original magnitude when the needle was fully retracted (see Fig. 3a). This behaviour is expected since a tensile stress (downward bending) serves to attract additional electrons and increase the 2DEG density, while a compressive stress (upward bending) depletes it. For device 2, a similar 1 μm bending of the cantilever yielded 33% change in *R*_DS_ (Fig. 3b) when *V*_GS_=−3.0 V and *V*_DS_=30 mV. Both devices showed very good repeatability for step bending responses, as shown for device 2 in Fig. 3b. The gauge factor (GF), which is a very important metric for strain (deflection) sensitivity, was calculated from the bending results using the relation^28^ GF=(Δ*R*_DS_/*R*_DS_)/*ɛ*_av_, where Δ*R*_DS_ is the change in *R*_DS_ due to bending, Δ*R*_DS_=*R*_final_−*R*_initial_ and *R*_DS_=*R*_final_, and *ɛ*_av_ is the strain in the HFET channel averaged over its width. The average strain (*ɛ*_av_) was determined from finite element simulations using COMSOL software (see Supplementary Fig. 4), which yielded *ɛ*_av_=4.477 × 10^−5^ for 1 μm bending of the free end of the microcantilever. The calculated GFs for the devices 1 and 2 were 8,700 and 7,300, respectively, for *V*_GS_=−3.0 V. The former is ∼91 and 3 times higher than the best GF values reported for Si piezoresistors (95)^15^ and single wall carbon nanotube-based strain sensors (2,900)^1^.

A plot of GF against *V*_GS_ is shown in the inset of Fig. 3a, where GF is found to decrease monotonically with the increase in *V*_GS_ (less negative values). From Supplementary Equation 1 and discussions in Supplementary Note 1, it follows that for gate voltage bias close to the threshold voltage,


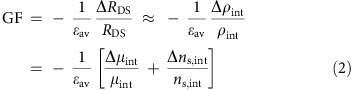


Since increase in *V*_GS_ causes both *n*_s,int_ and *μ*_int_ to increase (see earlier discussions), it is clear from equation (2) that GF should monotonically decrease with increase in *V*_GS_, and *vice versa*. Maintaining *V*_GS_=−3 V, the cantilevers were deflected by the same magnitude of 1 μm in 100-nm bending steps. A fairly consistent step change in *R*_DS_ of ∼3.8% per step (∼500 Ω nm^−1^) was observed for device 1 (see Fig. 3c) and ∼3.2% per step (∼410 Ω nm^−1^) for device 2. Good linearity in response over a larger dynamic bending range, from 100 to 10 μm, was also observed for both devices as shown in Fig. 3d. The direct current (dc) power consumption (*P*_DC_) calculated for both devices using the relation 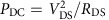, were found to be 1.36 nW and 0.82 nW, with *V*_DS_=30 mV and *R*_DS_=660 kΩ and 1.1 MΩ (in relaxed state), respectively. A separate experiment was conducted to determine the measurement repeatability over multiple deflection cycles, which revealed very consistent deflection-sensing performance of these devices (see Supplementary Fig. 5).

### Transduction of dynamic deflection

**Figure 4:**
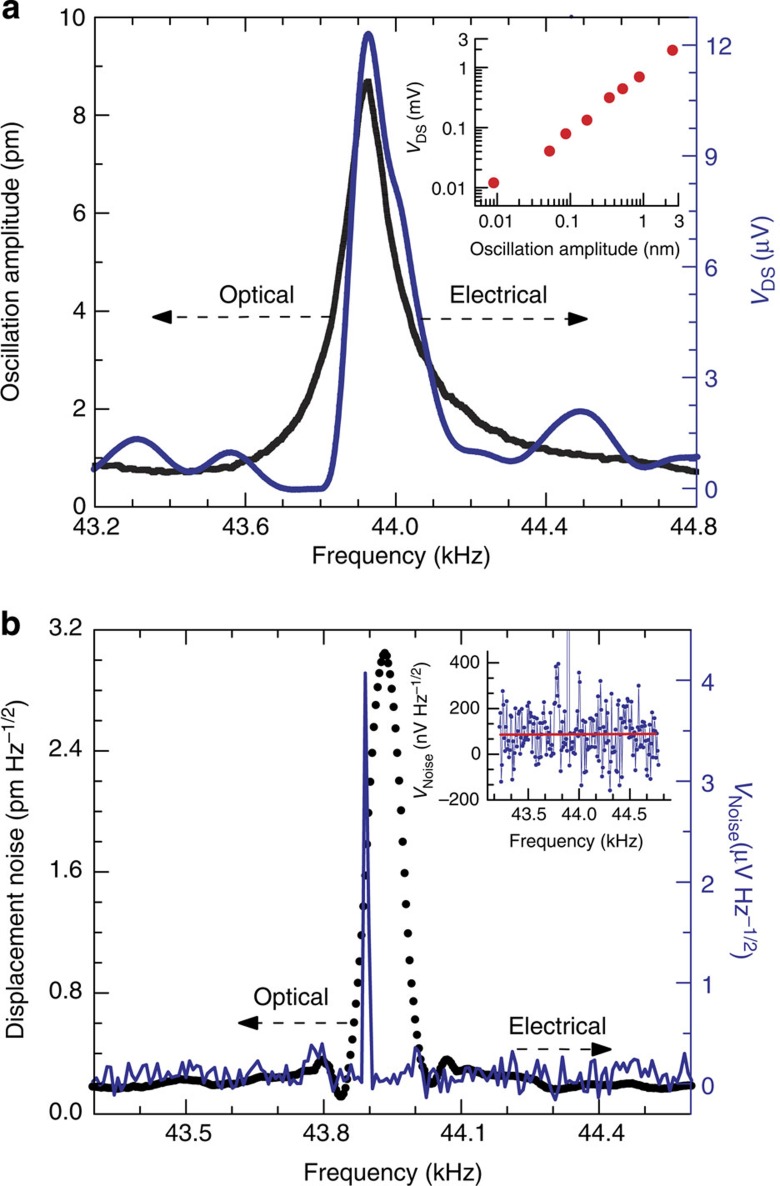
Sensing of Pico-scale dynamic deflection (HFET and optical readouts with or without external excitations). (**a**) Microcantilever 1 resonance curves measured simultaneously by both electrical and optical transduction methods show a resonant frequency of 43.934 kHz. An oscillation amplitude of 8.7 pm (from laser vibrometer) for the cantilever 1 corresponds to Δ*V*_DS_=12.36 μV (from HFET 1). The ac voltage applied to piezo oscillator was 10 mV (r.m.s.). Inset shows a linear response of the HFET for oscillation amplitudes varying over the range of 8.7 pm to 3 nm by gradual increase in the excitation voltage to the piezo from 10 to 250 mV. For the measurements, a constant bias current of *I*_DS_=100 μA, and a gate voltage of *V*_GS_=−2.2 V was maintained. (**b**) Noise spectra of microcantilever 1 exhibit a displacement noise of 3.04 pm Hz^−1/2^ (from laser vibrometer, black line), which corresponds to 4.07 μV Hz^−1/2^ noise measured by HFET 1 (blue line). Inset shows a plot of the off-resonance voltage noise due to the thermal oscillation of the microcantilever 1, with the average noise level marked by the red line. HFET 1 was biased at *V*_DS_=0.5 V and *V*_GS_=−2.2 V.

To investigate if externally excited microcantilever oscillations (that is, using a piezo-chip) could be transduced efficiently by the embedded HFET with very high sensitivity, a commercially available miniature piezo actuator (5 × 5 × 2 mm^3^) bought from PI (Model# PL 055.31) was placed in firm contact with the top surface of the DIP chip carrier (see Supplementary Fig. 6a), and vibrated by applying a variable frequency sinusoidal alternating current (ac) voltage to it from a lock-in amplifier (Model# SR850, Stanford Research Systems). For electronic transduction of the cantilever oscillations, a constant drain–source current (in the range of 1–100 μA) was maintained, and an appropriate gate bias was applied using the SMU. The amplitude of the ac voltage generated across the drain and source (Δ*V*_DS_) of the HFET due to cantilever oscillations was measured using the lock-in amplifier. For independent verification, simultaneous optical transduction of the cantilever oscillations was carried out using a laser Doppler vibrometer (Model# MSA 500, Polytec Inc.) as shown in Supplementary Fig. 6b. The laser spot of the vibrometer was focused on the gold pad at the tip of the cantilever for better reflection, as GaN microcantilever is transparent to the wavelength (635 nm) of the laser beam. Incidentally, this underlines the utility of the piezotransistive transduction compared with the optical one, which suffers from issues like material transparency and limited or diffused reflection. Since Δ*V*_DS_=*I*_DS_ × Δ*R*_DS_, to increase Δ*V*_DS_ (and correspondingly increase the sensitivity) *I*_DS_ and/or Δ*R*_DS_ need to increase. While *I*_DS_ is set by the user, Δ*R*_DS_ depends on the magnitude of the strain induced by the oscillation amplitude of the microcantilever, which can be tuned according to equation (1). Thus a more negative *V*_GS_ would result in lower *n*_s_ and a higher Δ*V*_DS_. Details on the optimization process are included in Supplementary Note 2 (see Supplementary Fig. 11). With *V*_GS_=-2.2 V and *I*_DS_=100 μA, oscillation of the microcantilever 1 in open air was observed to produce a Δ*V*_DS_=12.36 μV at the resonance frequency *f*_0_=43.934 kHz with a quality factor *Q*=230 (see Fig. 4a). Simultaneous optical measurement (using laser Doppler vibrometer) of cantilever oscillation showed its amplitude to be 8.7 pm. Different oscillation amplitudes of the cantilever 1, ranging from 8.7 pm to 2.5 nm (caused by ac excitation voltage varying from 10 to 250 mV applied to the piezo-chip), showed a linear of Δ*V*_DS_ as can be seen in the inset of Fig. 4a. For device 2, an oscillation amplitude of 17 pm at its resonance frequency of 46.4 kHz (*Q*∼350), yielded Δ*V*_DS_=23 μV as shown in Supplementary Fig. 7. For both the cantilevers, *f*_0_ and *Q* determined from the electrical and the optical spectra match closely, indicating the reliability of the electrically transduced signal. The dc power consumptions (*P*_DC_) calculated for the devices (using 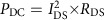) were 51 μW and 56 μW, respectively, with *I*_DS_=100 μA and *R*_DS_=5 kΩ and 6 kΩ, respectively (from the *I*_DS_–*V*_DS_ characteristics of the device 1 and 2).

The ability to electrically transduce the thermal noise spectra of a microcantilever is an important benchmark for deflection sensitivity. Due to very high deflection sensitivity of the III-Nitride piezotransistive microcantilevers, they could be used to electrically transduce their own thermal oscillations for the first time, which so far has only been possible through optical transduction method. To measure the thermal noise spectra, a constant *V*_DS_ of 0.5 V was applied to the HFET, and the change in *I*_DS_ was amplified using a low-noise current preamplifier (Model# SR570) whose output was connected to a dynamic signal analyzer (Model# SR785, Stanford research Systems). The sensitivity of the preamplifier was set at 1 mA V^−1^ and the recorded data was averaged three times. The thermal oscillation spectra obtained electrically from the HFET, and optically from the laser Doppler vibrometer (for comparison), are shown together in Fig. 4b. The peak voltage magnitude of 4.07 μV corresponds to a peak amplitude of 3.04 pm measured using the laser Doppler vibrometer.

The transduction gain or displacement responsivity of the cantilever can be calculated from the electrical and optical signal amplitudes at resonance as 4.07 μV/2.84 pm=1.43 nV fm^−1^, since the off-resonance noise is negligible. Using the responsivity of 1.43 nV fm^−1^, the electrical signal is calculated as 12.44 μV corresponding to the peak oscillation amplitude of 8.7 pm, which is in excellent agreement with the experimentally observed electrical peak amplitude of 12.36 μV, as shown in Fig. 4a. From the off-resonance voltage noise data, as shown in the inset of Fig. 4b, the noise spectral density can be measured as 86 nV Hz^−1/2^, which is much higher than the Johnson noise of 9.12 nV Hz^−1/2^ (calculated in the Supplementary Note 3) and likely incorporates noise from other sources, that is, from current preamplifiers, dynamic signal analyzers, cables, and so on. Nonetheless, the off-resonance noise-limited displacement resolution can be estimated from the responsivity as 86 nV/1.43 nV fm^−1^=60.14 fm, for a measurement bandwidth of 1 Hz. Following a similar approach, for device 2, the displacement responsivity was found to be 1.3 nV fm^−1^, and the noise-limited displacement resolution was found to be 3.42 pm (at resonance) and 105.2 fm (off-resonance including measurement noise) at a bandwidth of 1 Hz. It is quite significant to note that the displacement responsivities of both devices (1.43 and 1.3 nV fm^−1^) are ∼30 times higher than that previously demonstrated using a nanocantilever (0.04 nV fm^−1^)^2^, while the noise-limited resolutions at 1 Hz bandwidth (60.14 and 105.2 fm) are very comparable to the nanocantilever (39 fm)^2^ and almost 4 orders higher than similar sized microcantilevers (0.5 nm Hz^−1/2^)^2^ with comparable *f*_0_. We would like to point out here that for the first time we have demonstrated femtometre level displacement resolution with complete electrical displacement transduction for a microcantilever operating <100 kHz. The optically measured resonance amplitude noise of 3.04 pm Hz^−1/2^ closely matches with that due to thermomechanical noise of 2.84 pm Hz^−1/2^.

### Transduction of acoustic wave

**Figure 5:**
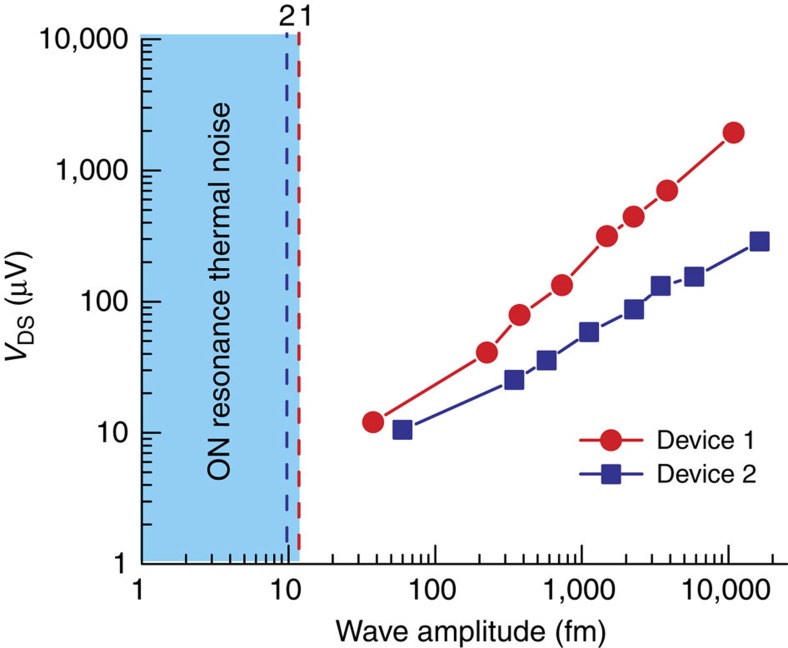
Relation between HFET readouts and external acoustic excitation. Electrical responses of HFETs 1 and 2 corresponding to microcantilever oscillation amplitude variation caused by variation in external acoustic excitation (over a range of 37.8 fm to 10 pm) produced by the piezo-chip. The dashed lines parallel to the *y*-axis represent the on-resonance thermomechanical noise-limited excitation amplitudes of 12.35 fm Hz^−1/2^ and 9.77 fm Hz^−1/2^, respectively. The responsivity (sensitivity) of the devices in transducing surface wave to electrical voltage can be estimated from the slope of the linear responses as 170 nV fm^−1^ and 60 nV fm^−1^, for device 1 and 2, respectively.

The amplitude of the mechanical vibrations (that is, the driving amplitude of the acoustic wave *A*_d_) acting on the microcantilever can be determined from the oscillation amplitude of the cantilever, *A*_0_ as, *A*_d_=*A*_0_/*Q*. For device 1, the wave amplitude can be estimated as 37.8 fm (=8.7 pm/230, refer to Fig. 4a), while the thermomechanical noise-limited driving amplitude was determined as 12.35 fm Hz^−1/2^ (=2.84 pm Hz^−1/2^/230). For device 2, the wave amplitude was estimated as 48.57 fm (refer Supplementary Fig. 7) and the noise-limited driving amplitude was found to be 9.77 fm Hz^−1/2^. In our experiment, the piezo excitation was varied to generate different driving amplitude levels over a range of 37.8 fm–10 pm, and both the devices showed linear response over this large range as shown in Fig. 5. The displacement sensitivities (for detecting the driving acoustic wave) of devices 1 and 2 were calculated from the slope of the linear responses in Fig. 5, as 170 nV fm^−1^ and 60 nV fm^−1^, respectively. The off-resonance noises (which includes Johnson noise and equipment noise) were measured as 86 nV Hz^−1/2^ and 136.76 nV Hz^−1/2^ for devices 1 and 2, respectively, and the corresponding noise-limited displacement resolutions were found as 0.51 fm Hz^−1/2^ (86 nV Hz^−1/2^/170 nV fm^−1^) and 2.3 fm Hz^−1/2^ (136.76 nV Hz^−1/2^/60 nV fm^−1^). The vertical dashed lines in Fig. 5 show the noise limits for surface wave detection. From the above discussion, it is quite clear that the devices are capable of measuring surface wave amplitudes in the tens of femtometre range, which is better than the optical transduction technique^29^. The measured wave amplitudes for device 1 was slightly lower (by ∼22.1%) as the excitation source was located farther compared with that to device 2 (see Fig. 1b).

To verify that the cantilever excitation amplitude is really in the femtoscale, the vibration amplitude of the top surface of the piezo-chip was measured using the laser Doppler vibrometer for various excitation voltages applied to the piezo (10–250 mV), and the results are shown in Supplementary Fig. 8. For the lowest oscillation amplitude of the piezo-chip of 400 fm, the microcantilever oscillation amplitude was found to be 8.7 pm (and Δ*V*_DS_=12.36 μV), which would result from a surface wave excitation amplitude of 37.8 fm (=8.7 pm/230). More than 10-fold reduction in the exciting wave amplitude compared with the piezo vibration can be caused by attenuation, damping introduced at the bottom surface of the piezo-chip due to solid contact to the ceramic and acoustic impedance mismatch of different media as encountered by the propagating wave.

**Figure 6:**
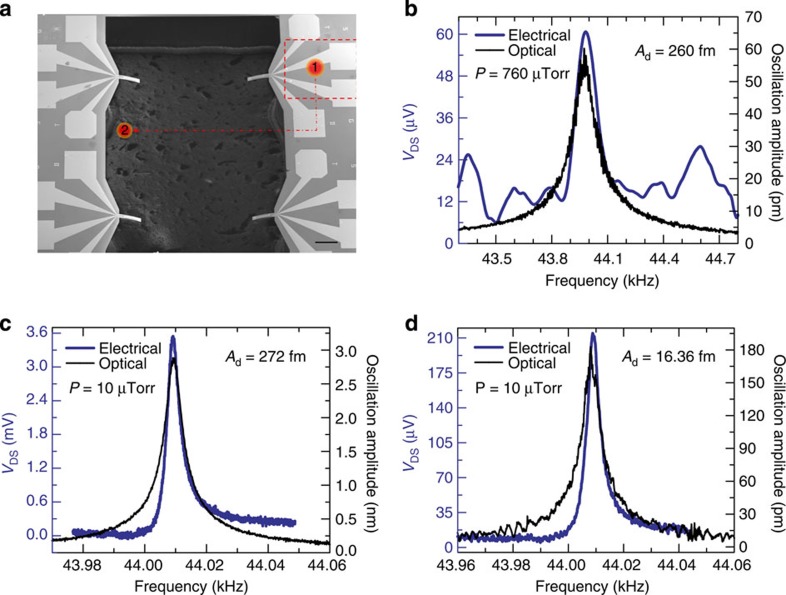
Detection of femtoscale acoustic excitations (HFET and optical readouts). (**a**) SEM image of four microcantilevers showing the relative positions of the laser spots (∼50-μm diameter) used for photoacoustic excitation (scale bar, 500 μm). The laser (wavelength of 790 nm) was pulsed with a variable frequency (43–45 kHz) sinusoidal signal. (**b**) Frequency response of HFET 1 transduced both optically and electrically when the laser spot was focused at position 1 (measurement conducted in air). The driving amplitude (*A*_d_) of the surface wave was determined to be 260 fm (oscillation amplitude, *A*_0_=60 pm divided by the quality factor (≈230) of microcantilever 1). (**c**) Frequency response of HFET 1 obtained with the laser spot at position 1 under 10 μtorr pressure (with *A*_d_ calculated as 272 fm). (**d**) Frequency response of HFET 1 when the laser spot was at position 2 at 10 μtorr, which yielded *A*_d_=16.36 fm, and corresponding Δ*V*_DS_=71.9 μV. The HFET was biased at *V*_DS_=0.5 V and *V*_GS_=−2.2 V as before.

To further investigate the femtoscale displacement transduction by the HFET, we conducted a separate set of experiments in ambient and high vacuum (10 μtorr), where we used photoacoustic excitation of the microcantilever 1 using a near infrared (IR) pulsed laser (wavelength of 790 nm, WorldStar Technologies, Inc.). The energy of the laser pulse is absorbed by the Si substrate (since GaN is transparent to 790 nm), and the photothermal effect generates an acoustic wave which propagates through Si substrate and reaches the cantilever to cause the oscillations. With the laser focused on a ∼50 μm diameter spot (marked as position 1 in Fig. 6a), the HFET yielded Δ*V*_DS_=61 μV, while the cantilever oscillation amplitude was found to be 60 pm (see Fig. 6b). The amplitude of the periodic excitation (due to the acoustic wave) can then be calculated as 260 fm assuming the same quality factor of 230. Keeping the laser focused on the same spot, the pressure was reduced to 10 μtorr, which caused the Q-factor to increase significantly to 11,000 (see Supplementary Fig. 9), which greatly enhanced the oscillation amplitude of the microcantilever to 3 nm, as shown in Fig. 6c. Once again, a very good match is observed between the electrical and optical response curves, with the electrical signal Δ*V*_DS_=3.6 mV corresponding to 3 nm amplitude measured by the vibrometer. The amplitude of the surface wave was estimated as 272 fm (=3 nm/11,000), which is close to that estimated under ambient conditions, clearly indicating that high vacuum does not affect the acoustic wave propagation, as expected. When the laser was focused on an epoxy layer in the DIP cavity (shown as position 2 in Fig. 6a), the microcantilever oscillation amplitude decreased significantly to 60 pm, which indicates that the exciting wave amplitude was 5.45 fm (=60 pm/11,000). The electrical signal corresponding to the amplitude was measured as Δ*V*_DS_=71.9 μV as shown in Fig. 6d, which yields a responsivity of ∼1.2 μV pm^−1^ (for transducing surface acoustic wave). At position 2, the laser power was absorbed almost completely by the epoxy used to glue the Si substrate to the bottom (Au coated) DIP. The acoustic wave generated by the epoxy is very weak but due to the high quality factor of 11,000 of the GaN microcantilever in vacuum faint acoustic wave of 5.45 fm amplitude was possible to transduce with a high voltage responsivity of 12.96 μV fm^−1^. However, no cantilever oscillation was observed in atmospheric pressure when the laser was focused at the same spot, although similar exciting wave amplitude of 5.45 fm as in high vacuum is expected (see above discussions). This is because, in atmospheric pressure the quality factor of the cantilever is much reduced (230), which results in the microcantilever oscillation amplitude of 1.25 pm that is smaller than the thermomechanical noise of the cantilever of 2.84 pm Hz^−1/2^ at nominal 1-Hz frequency bandwidth. Therefore, only thermal oscillations of the cantilever could be observed in air with the laser focused on position 2. In vacuum, the calculated thermomechanical noise of the microcantilever increased to 19.67 pm Hz^−1/2^ (calculated using *Q*=11,000, *f*_0_=44,010 Hz and Δ*f*=1 Hz, which matches well with 19.95 pm Hz^−1/2^ observed from the optical measurement. This noise is, however, much smaller than the oscillation amplitude of the cantilever of 3 nm, which can thus be easily transduced both optically and electrically. Interestingly, the high quality factor in vacuum yields an ultralow displacement noise floor of 1.79 fm Hz^−1/2^ (=19.95 pm Hz^−1/2^/11,000), which puts the lower limit for detection of the exciting acoustic wave at 1.79 fm for a bandwidth of 1 Hz. Such extremely low detection limit can open up novel opportunities for surface wave-based photoacoustic analysis and detection using compact microscale sensors. The static and dynamic performances of both devices are summarized in Supplementary Table 1.

### Photoacoustic spectroscopy of analytes

**Figure 7:**
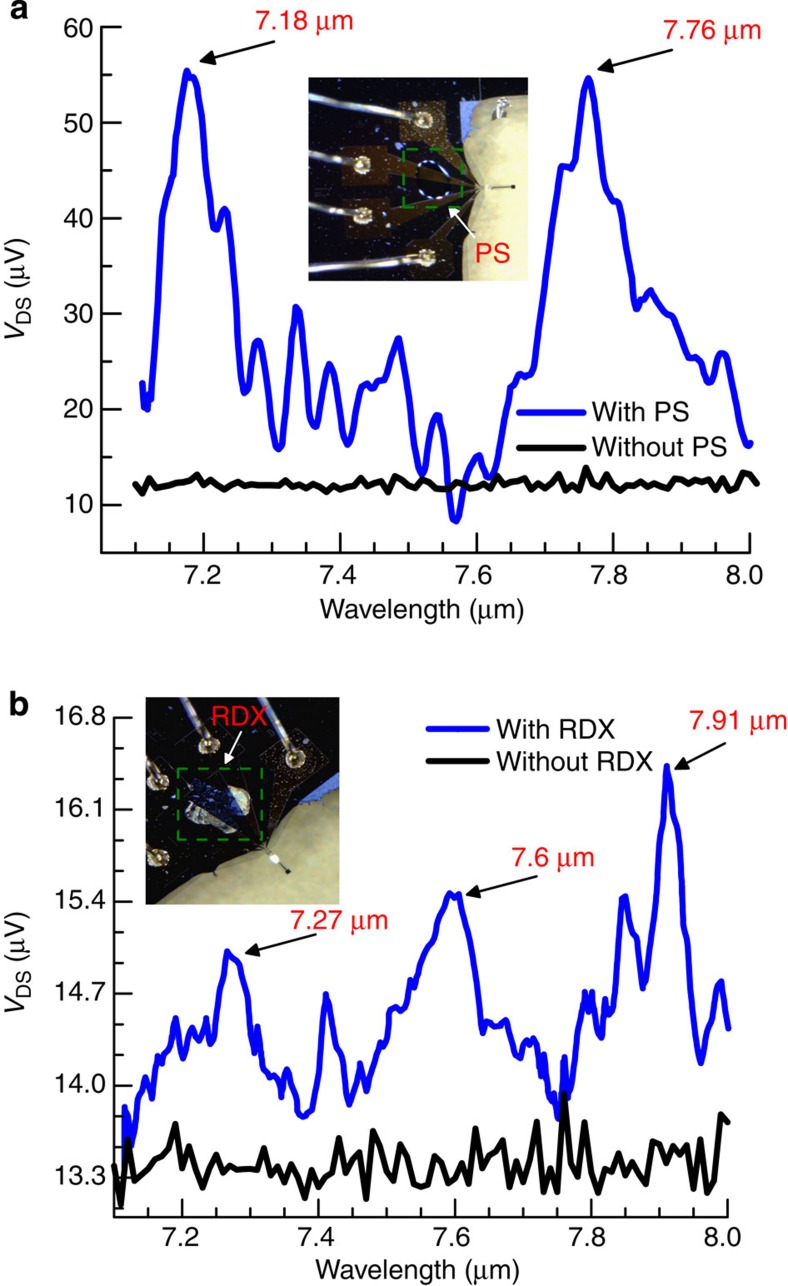
Photoacoustic spectroscopy of analytes with HFET readout. (**a**) Photoacoustic spectroscopy of polystyrene (PS) with piezotransistive transduction exhibiting two characteristic peaks at 7.18 and 7.76 μm. Inset shows the optical image of PS deposition near the base of the microcantilever 1. (**b**) Photoacoustic spectroscopy of RDX with piezotransistive transduction revealing three characteristic peaks at 7.27, 7.6 and 7.91 μm. Inset shows the optical image of the RDX deposition near the base of microcantilever 1.

The ultra-high sensitivity of the electrical transduction method to surface waves enabled us to perform unique detection of surface-deposited analytes through photoacoustic spectroscopy^30^,^31^. Two different analytes, polystyrene (PS) and research development explosive (RDX; chemical name: 1,3,5-trinitroperhydro-1,3,5-trazine), were chosen to demonstrate photoacoustic detection using these piezotransistive microcantilevers. These analytes were deposited near the base of a microcantilever (shown in the insets of Fig. 7a,b), and a tunable wavelength (*λ*=7.1–8.0 μm with a step size of 20 nm, 5 mW) mid-IR quantum cascade laser (Daylight Solutions, UT-8) was focused on them and pulsed at the resonance frequency of the microcantilever (43.93 kHz). The experimental set-up is shown in Supplementary Fig. 10. In our experiments, we initially deposited PS, which was later removed using a tweezer to deposit RDX at the same location. Approximately 300 nl of both the analytes were deposited using their standard solutions (1 mg ml^−1^) using a capillary glass tube and allowed to dry. The background signal was initially recorded from the HFET prior to analyte coating, using a lock-in amplifier, with the HFET biased at *V*_DS_=0.5 V, *V*_GS_=−2.2 V and *I*_DS_=100 μA. The HEFT output signal recorded after analyte coating was subtracted from the background signal recorded prior to coating, to obtain the final photoacoustic spectrum of the analytes. The microcantilever oscillation amplitude varied depending on the extent of IR absorption by the deposited analyte over the mid-IR wavelength range. The IR absorption peaks of PS are shown in Fig. 7a, which closely matches the representative absorption peaks reported earlier^32^. The IR absorption signature peaks of RDX (∼300 ng) are shown in Fig. 7b, which are also in excellent agreement with previous reports^33^,^34^. From the magnitudes of Δ*V*_DS_ corresponding to the signature peaks of PS and RDX, the amplitude of the exciting wave can be determined to be in the femtoscale range (∼100–300 fm) using the amplitude–Δ*V*_DS_ correlation in Fig. 5. We would like to point out several novel aspects of the sensing methodology. First, for the first time we have demonstrated unique electrical detection of small amount of surface-deposited analyte using photoacoustic spectroscopy. Second, and perhaps more significantly, the detection has been possible with complete electrical deflection transduction due to the development of novel and highly sensitive piezotransistive microcantilevers operating in the tens of kilohertz range. Third, the microcantilever does not need to be modified in any way, and thus can remain pristine and be used repetitively. Fourth, the microcantilever can be enclosed in vacuum, which would further enhance the detection sensitivity enabling detection pictograms of analytes. Finally, an array of piezotransistive microcantilevers can be easily fabricated, which would enable rapid and simultaneous detection of a large variety of analytes with a microscale footprint.

### Comparison with state-of-the-art

**Figure 8:**
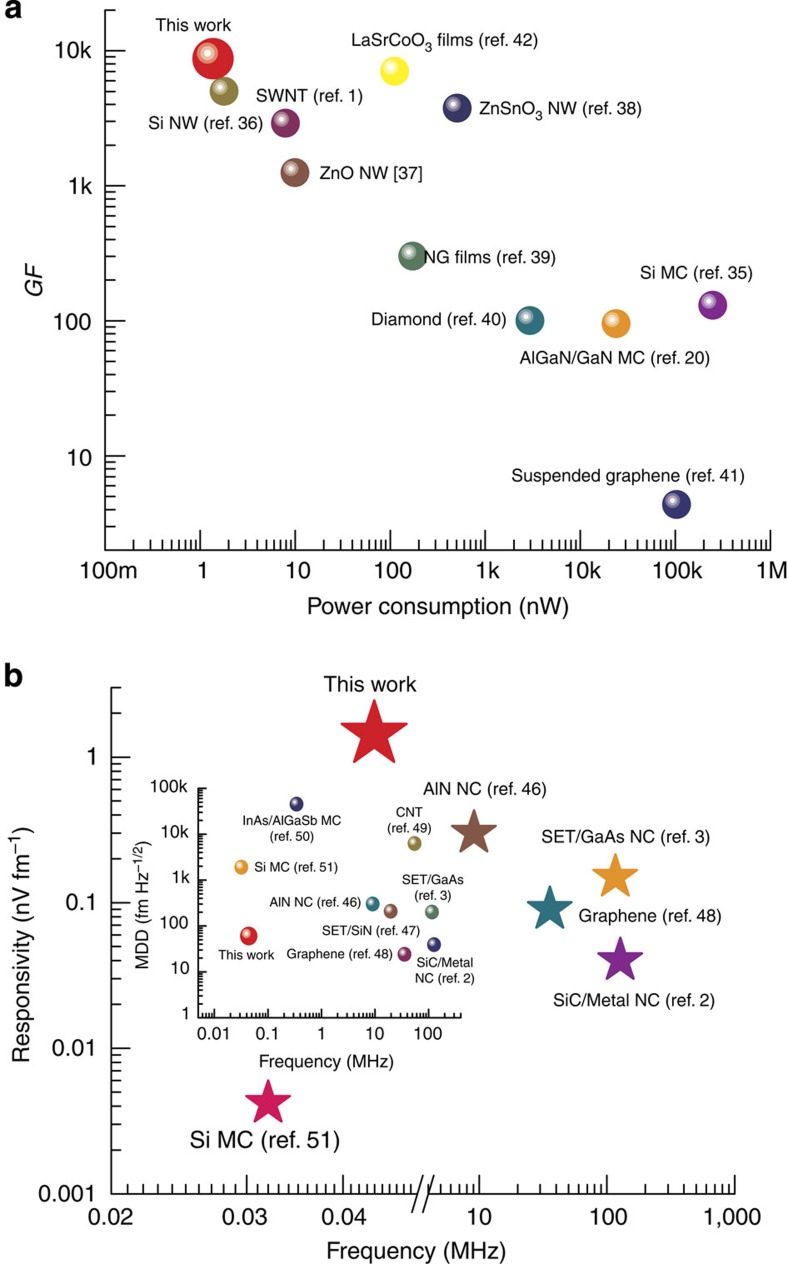
Comparisons between Piezotransistor and state-of-the-art technologies. (**a**) Best reported gauge factors (GFs) for different sensor technologies plotted against device power consumption. The highest GF (8,700), considering all technologies reported so far, is demonstrated by the piezotransistive microcantilever presented in this work, which also consumes the lowest power of 1.36 nW. (**b**) Plot of the best reported responsivities (in transducing mechanical oscillation of suspended structures) versus frequency also demonstrates the outstanding performance (VR=1.43 nV fm^−1^) of our device, which clearly addresses a technology void in highly sensitive mechanical excitation detection in <100 kHz range. Inset shows the minimum detectable displacement (MDD) of various technologies (measured off-resonance) plotted against frequency. The piezotransistive microcantilever yields an MDD of 2.84 pm Hz^−1/2^ at resonance, which goes down to a value of 60.14 fm Hz^−1/2^ off-resonance, which is comparable with the best MDD values reported so far considering all technologies over all frequency ranges.

To put the performance of our microcantilever in perspective, we have compared the sensitivity and power consumption of various piezoresistive sensor technologies. While the best reported GF for Si piezoresistors, the traditional workhorse for strain sensing, has been ∼200 (ref. 1) for single crystalline thin film (and 130 for microcantilevers^35^), nanoscale piezoresistors of Si^36^, CNT^1^, ZnO^37^ and ZnSnO_3_ (ref. 38) have all yielded high GFs >1,000. Recently, grapheme- (film^39^ and suspended^40^) and diamond-based^41^ piezoresistors have been investigated although their reported GFs are comparatively lower (<300). Till date, one of the highest GFs has been demonstrated by LaSrCoO_3_ of ∼7,000 (ref. 42), but is suffers from strong non-linearity in response. In addition, the aforementioned piezoresistors often require a controlled environment for operation (high vacuum and/or low temperature), and face challenges in terms of repeatability and consistency (especially those based on nanoscale materials). Only a handful of studies have been reported on the piezoresistive properties of 2DEG formed at the AlGaN/GaN interface^20^,^23^,^43^,^44^,^45^. In the earlier works, the III-Nitride epilayers studied were grown on sapphire substrate^23^,^43^,^44^,^45^, which could not be etched, therefore application of strain on the epilayers was cumbersome and difficult to estimate accurately. In addition, the sensing elements were in the form of simple AlGaN/GaN piezoresistors where gate control was either absent or unoptimized. The combined effect of these two factors led to GF values <100 (refs 20, 23, 43, 44, 45). Our design takes advantage of high quality epilayers that are currently synthesized on Si wafers, and utilizes and optimized biasing schemes for the HFET at the base of a microcantilever, resulting in ultra-high sensitivity. Our proposed piezotransistive microcantilevers have demonstrated, to the best of our knowledge, the highest GF till date of 8,700 (in open ambient) considering all material systems, while consuming a very low power of 1.36 nW. The GF and power consumption of various technologies are compared in Fig. 8a, which shows a general trend of increasing GF with reduction in power consumption.

Although the transducers mentioned above show very high GF, their frequency response have not been reported using non-optical transduction methods. Piezoelectric (AlN)^46^ and semiconductor/metal^2^ nanocantilevers, and single electron transistor-based doubly clamped nanoscale beams made of GaAs^3^ and SiN^47^ have shown great responses with minimum detectable displacement (MDD) <300 fm Hz^−1/2^ (in atmospheric ambient). Recently, suspended graphene-48 and CNT-based^49^ NEMS has demonstrated a low value of MDD (35.8 fm Hz^−1/2^ and 6.25 pm Hz^−1/2^, respectively), but only under high vacuum with significant enhancement in their low quality factor. InAs/AlGaSb heterostructure-based displacement sensor has also been demonstrated^50^, however, the MDD was found to be quite high (∼45 pm Hz^−1/2^), which is even much higher than that reported for Si piezoresistors (∼1.9 pm Hz^−1/2^)^51^ suffering from very low responsivity (4.2 pV fm^−1^) in the similar frequency regime (32 kHz)^51^. On the other hand, our microcantilevers have demonstrated a much superior MDD of 12.35 fm Hz^−1/2^ in open ambient, and 1.79 fm Hz^−1/2^ in vacuum with a very high responsivity of 1.43 nV fm^−1^ in air. Recently, there have been some reports on resonant body transistors for high frequency acoustic wave detection and filtering applications in mega to gigahertz range^52^,^53^,^54^, however, none of them have reported the detection sensitivity or MDD for acoustic vibrations. Figure 8b and its inset compare the responsivity and MDD of various deflection sensor technologies as a function of frequency.

## Discussion

In summary, a piezotransistive GaN microcatilever embedded with highly sensitive AlGaN/GaN HFET deflection sensor has been demonstrated. Ultra-high values of GF (8,700) and responsivity (1.43 nV fm^−1^) have been measured, which are highest reported till date, considering all technologies. The outstanding sensitivity of the microcantilevers, verified through analytical calculations and laser vibrometry measurements, enabled them to detect femtoscale acoustic wave excitation amplitudes, and demonstrate for the first time electrically transduced photoacoustic detection of nanogram-level surface-deposited analytes. These microcantilevers, which are operable over a broad range of frequencies spanning from dc to several tens of kilohertz address a technological void, and can have a transformative impact on a large variety of fields requiring ultrasensitive measurements, including scanning probe-based imaging and MEMS/NEMS-sensing applications.

## Methods

### Step bending experiments

In these experiments, we bent the microcantilever by contacting the cantilever edge using a needle with a tip diameter of 12 μm. The needle was attached to a nanopositioner bought from Physic Instrument and controlled using Labview software installed in a computer. A dual channel SMU from Keithley was used to apply voltage to the HFET, and both the current and resistance of the HFET were measured using the same SMU. Details on the HEFT biasing scheme have been included in Supplementary Note 2. The geometry (length and width of the cantilever) was determined from COMSOL-based simulations to obtain a resonance frequency in the range of 40-50 kHz, which is typically used for photoacoustic spectroscopy in the ultrasonic range. The resonance frequency from COMSOL simulation typically matches well with that obtained from analytical formulations for rectangular microcantilevers. While determining the dimensions of the gate, source and drain metal contacts and their positions, photolithographic feature size limitation and its possible impact on the overall device yield were also carefully considered. Apart from the conventional source, drain and gate contacts of the HFET, there is an additional contact for electrostatic actuation of the microcantilever, which was not used in this study.

### Dynamic bending experiments

For this experiment, commercially available miniature piezo actuator (5 × 5 × 2 mm) bought from PI was placed in firm contact with the top surface of the DIP chip carrier, and the piezo-chip was vibrated by applying a sinusoidal voltage to the piezo-chip from a lock-in amplifier. To measure the electrical signal produced by the oscillations, a constant current (1–100 μA) was passed through the HFET, and an appropriate gate bias was provided using the SMU. The amplitude of the ac component of the voltage across the drain and source of the HFET was measured using the lock-in amplifier.

For measurement of the thermal noise spectra of the cantilever, a constant voltage of 0.5 V was applied to the HFET and the drain current was amplified using a low-noise current preamplifier, which was then connected to a dynamic signal analyzer. The sensitivity of the preamplifier was set at 1 mA V^−1^, and no other signal enhancement was performed. In the dynamic signal analyzer, data were recorded with 10 times averaging. Optical measurement of both the thermal noise spectra and the dynamic oscillations induced by the piezo-chip, were carried out using a laser Doppler vibrometer. The *f*_0_ observed from the electrically transduced resonance curve differs by ∼34 Hz when compared with the optically transduced one, unlike in Fig. 4a, where they match closely. This can be explained by a change in cantilever surface conditions caused by a change in environment and time lapse, since the measurements involving electrical transduction of the thermal oscillations were performed in our laboratory at the University of South Carolina ∼1 month after the optical measurements were performed at the University of Alberta in Canada. The change in surface conditions may also be partially responsible for the difference in quality factor observed between the two resonance curves. On the other hand, the electrical and optical resonance curves under mechanical excitation (shown in Fig. 4a) were measured simultaneously at the University of Alberta, Canada, and thus match very well. The optically measured resonance amplitude noise of 3.04 pm Hz^−1/2^ closely matches with that due to thermomechanical noise of 2.84 pm Hz^−1/2^ with a difference of only 200 fm Hz^−1/2^ that is attributable to the noise level of the laser vibrometer as specified by the manufacturer (out-of-plane displacement resolution of <400 fm Hz^−1/2^). The off-resonance displacement noise was found to be 200 fm Hz^−1/2^ (Fig. 4b), which differs by ∼185 fm Hz^−1/2^ from the theoretical calculation of ∼15 fm Hz^−1/2^ considering only the thermomechanical noise for the laser vibrometer (as Johnson noise should not affect the optical measurements).

## Additional information

**How to cite this article:** Talukdar, A. *et al*. Piezotransistive transduction of femtoscale displacement for photoacoustic spectroscopy. *Nat. Commun.* 6:7885 doi: 10.1038/ncomms8885 (2015).

## Supplementary Material

Supplementary Information Supplementary Figures 1-11, Supplementary Table 1, Supplementary Notes 1-3 and Supplementary References
