# Supplementary material for: Piezotransistive transduction of femtoscale displacement for photoacoustic spectroscopy
Source: Nat Commun. 2015 Aug 10;6:7885. doi: 10.1038/ncomms8885 (PMC4918345; doi:10.1038/ncomms8885)
Supplement: Supplementary Information — Supplementary Figures 1-11, Supplementary Table 1, Supplementary Notes 1-3 and Supplementary References [file ncomms8885-s1.pdf]

Supplementary Information

Supplementary Figures

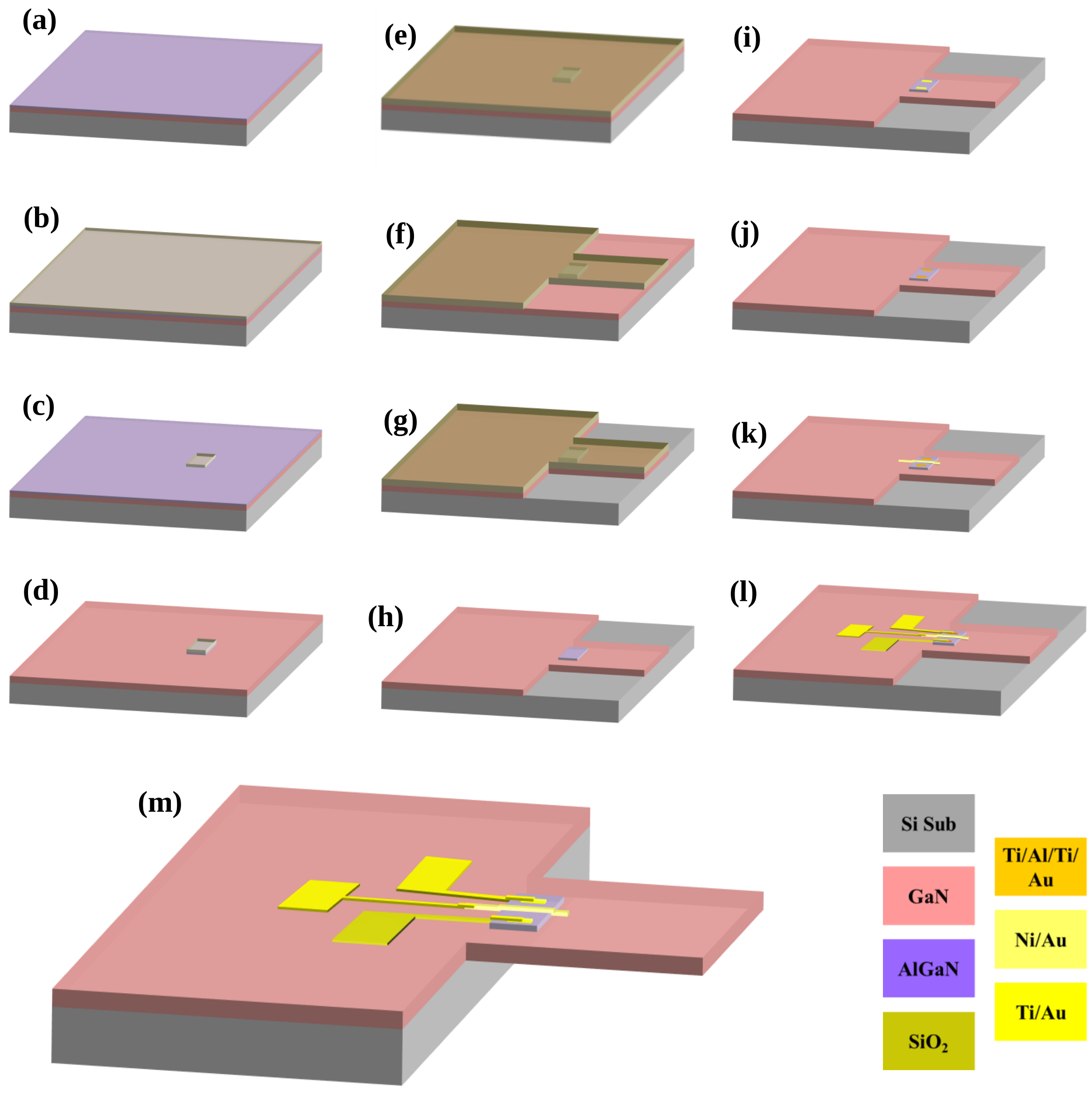

Supplementary Figure 1: Process flow diagram depicting the fabrication steps in realizing the GaN microcantilever with embedded AlGaN/GaN HFET at the base. (a) A diced sample (1.4 cm

by 1.4 cm square) with AlGaN/GaN epilayers grown on Si sample. Each diced piece incorporated a total of 16 similar microcantilevers in 4 pockets; (b) Plasma enhanced chemical vapour deposition (PECVD) of SiO_2 (300 - 400 nm); (c) Patterning the mesa layer with positive photo resist (SC1827) followed by dry etching of SiO_2 in Plasma Therm Inductively Coupled Plasma (ICP) tool; (d) ICP etching of ~200 nm of AlGaN and GaN (over etching was done to ensure complete isolation); (e) PECVD SiO_2 (1.2 µm) deposition; (f) Patterning of the microcantilever outline with negative photo resist (NR 71) and ICP etching of oxide; (g) ICP etching of GaN; (h) Complete oxide etching with BOE; (i) Patterning ohmic contact and e-beam deposition of Ti (20 nm)/Al (100 nm)/Ti (45 nm)/Au (55 nm) metal stack; (j) Rapid thermal annealing of ohmic contacts; (k) Patterning schottky gate contact and E-beam deposition of Ni (50 nm)/Au (200 nm) metal stack; (l) Patterning probe contact and e-beam deposition of Ti (25 nm)/Au (200 nm) metal stack (for clarity of representation the geometry of contact pads has been modified from the actual probe metal pad as shown in Fig. 1, and also the tip bias pad has been omitted); (m) through wafer Si etching from backside using Gas chopping etching process to release the microcantilever using SiO_2 as the hard mask, which was patterned with NR 71. The wafer was bought from DOWA Semiconductor Akita Co., Ltd. and the devices were fabricated in the Institute for Electronics and Nanotechnology (IEN) at the Georgia Institute of Technology.

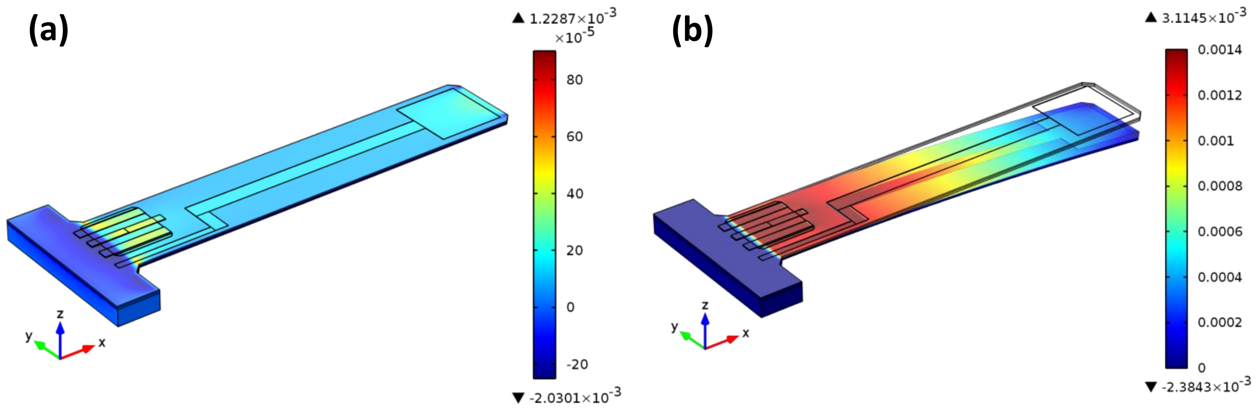

Supplementary Figure 2: COMSOL simulations showing stress distributions on the Microcantilever with (a) no bending, and (b) the tip bent by 30 μm. Strain values used for calculations were obtained from the simulated stress values and the Young's Modulus of GaN.

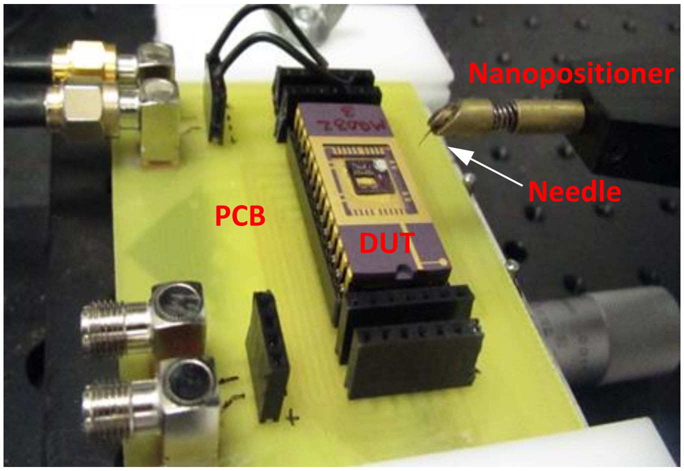

Supplementary Figure 3: Optical image of the experimental setup for step bending. A tungsten micro-needle (12 μm tip diameter) attached to a nanopositioner (controlled externally by a computer using labview) is used to bend the tip of the microcantilever. The cantilevers were wire bonded to a 28 pin DIP package which was inserted into a socket soldered on a printed circuit board for electrical readout.

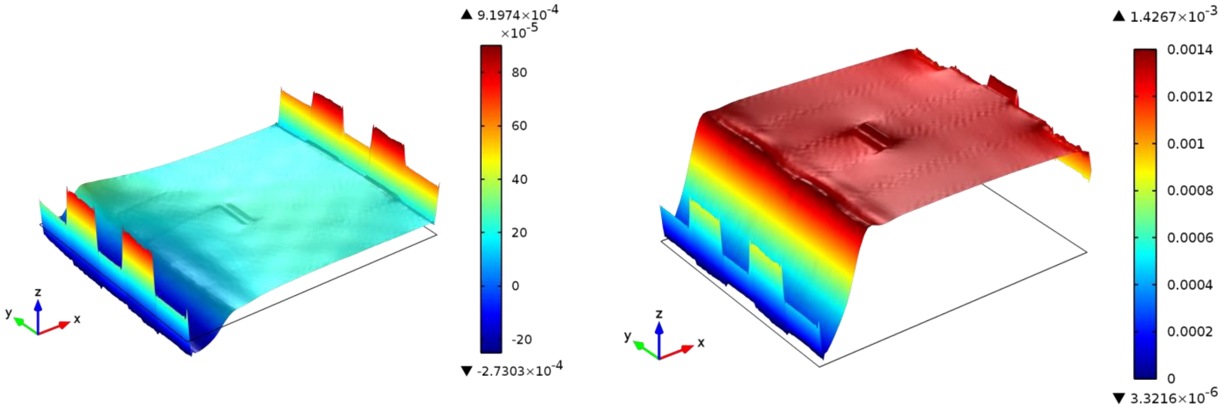

Supplementary Figure 4: Finite element simulations of stress distribution over the AlGaN mesa region which contains the HFET with (a) no bending and (b) the tip bent by 30 μm. Corresponding strain values were obtained from the simulations, where 30 μm bending yielded an average strain (along XY plane of the mesa) of 1.3431×10^{-3}.

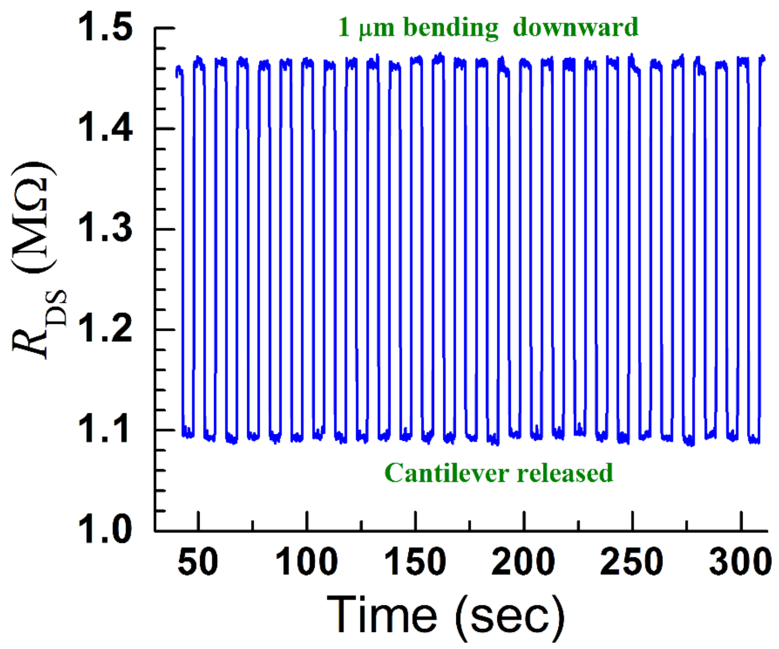

Supplementary Figure 5: Step bending performance of device 2 when the tip of the cantilever was bent 1 µm (downward) and released for multiple cycles (26 cycles are shown here). For each cycle, the cantilever was kept in the bent state for 5s and in the released state also for 5 s.

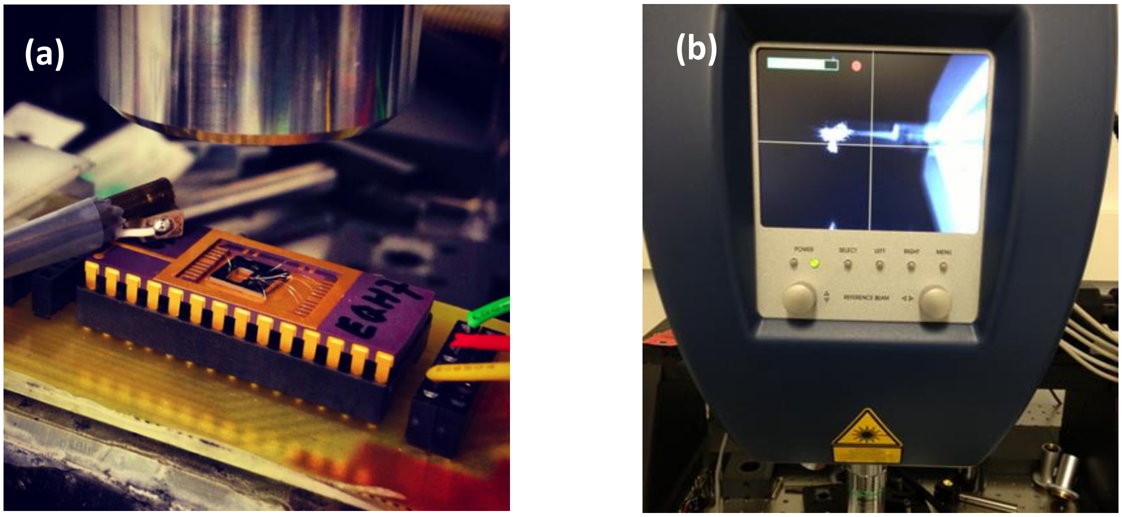

Supplementary Figure 6: (a) Experimental setup for simultaneous optical and electrical transduction of microcantilever deflections. A piezochip was held in contact with a DIP package, as seen in (a), to generate surface wave to oscillate the microcantilever. The electrical deflection transduction was performed by the HFET, with its signals read out by external instruments using a PCB. For optical deflection transduction, the whole package was placed under the lens of a laser vibrometer (Model# MSA500) which measured the oscillation amplitude. (b) The screen of the laser vibrometer shows the laser spot focused at the tip of the Microcantilever.

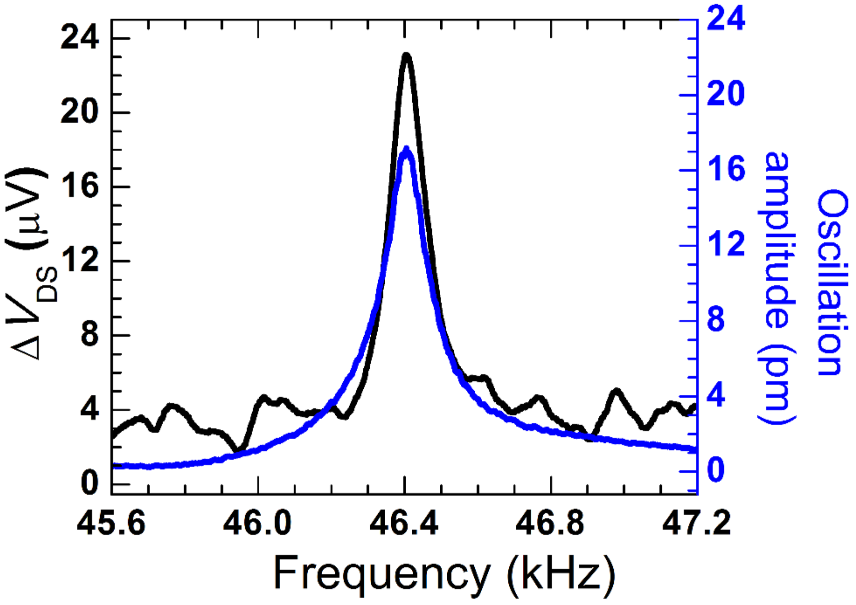

Supplementary Figure 7: Frequency response of the Microcantilever 2 obtained simultaneously using electrical and optical deflection transduction methods. A resonance frequency of 46.4 kHz with a Q ~350 is obtained from both measurements. An oscillation amplitude of 17 pm at the resonance frequency of the cantilever corresponds to ΔV_{DS} = 23 µV.

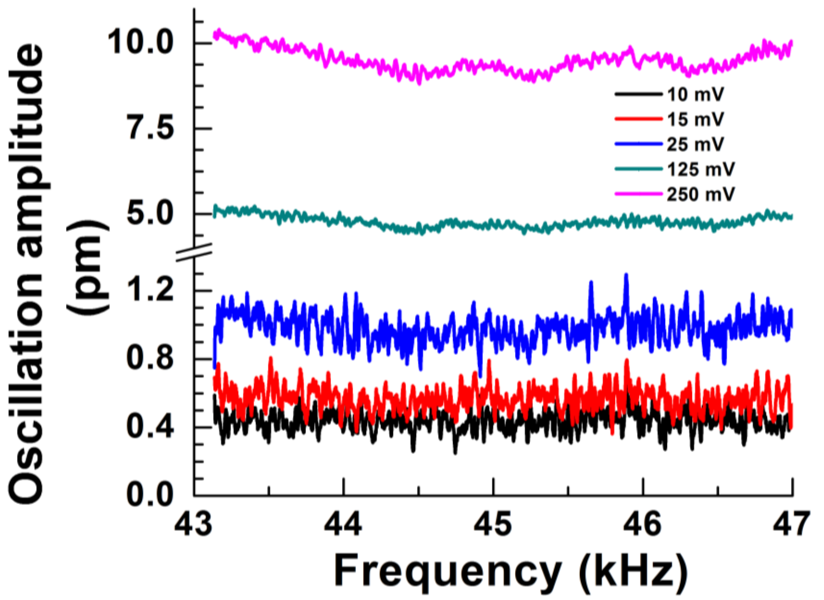

Supplementary Figure 8: Oscillation amplitude of the top surface (free end) of the piezochip measured using the laser vibrometer. The excitation voltage applied to the piezochip was varied from 10 to 250 mV, while the frequency was swept from 43 kHz to 47 kHz for each applied voltage. The piezochip had a flat frequency response in this frequency range, which ensures that a constant amplitude of the surface wave is generated (which excites microcantilever oscillations). Notably, the bottom plane (fixed to the surface) of the piezochip is expected to have much lower amplitude of vibration. Thus 10 mV is expected to produce an oscillation much less than 400 fm produced at the free surface. This indicates that the amplitude of exciting oscillation near the base of the microcantilever would be in the tens of fm range, as obtained from our measurements on the microcantilever.

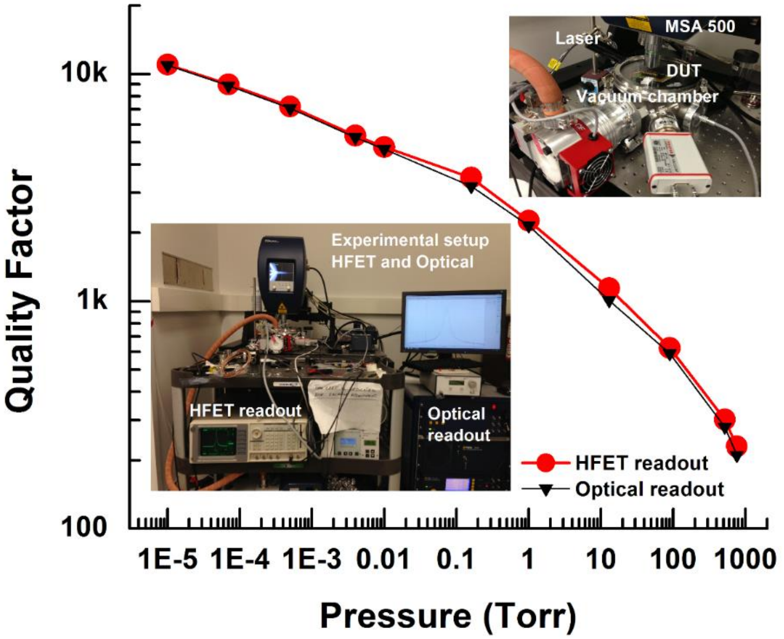

Supplementary Figure 9: The enhancement of the quality factor of microcantilever 1 when the pressure was changed from atmospheric pressure to 10 µTorr. Simultaneous optical (MSA 500) and HFET 1 readouts were obtained as shown in the setup in the bottom left inset. The resonance curves and quality factors from both readouts matched closely. Simultaneously measured sample resonance curves are shown in the computer monitor and the lock-in amplifier screen. The cantilever was excited with pulsed laser induced acoustic wave generated through laser absorption in the Si substrate. The top right inset shows a close up photograph of the vacuum chamber, device under test (DUT), lens of MSA 500 and the excitation laser mounted externally. The HFET's electrical connections were made through a specially designed 8-pin cable through a vacuum feedthrough. The HFET was biased using V_{DS} = 0.5 V, V_{GS} = -2.2 V and a constant I_{DS} = 100 µA. A mechanical roughing pump (not shown in the photo) was used to initially reduce the chamber pressure to 10 mTorr, when a turbo pump kicked-in reduce the chamber pressure further pump to µTorr range.

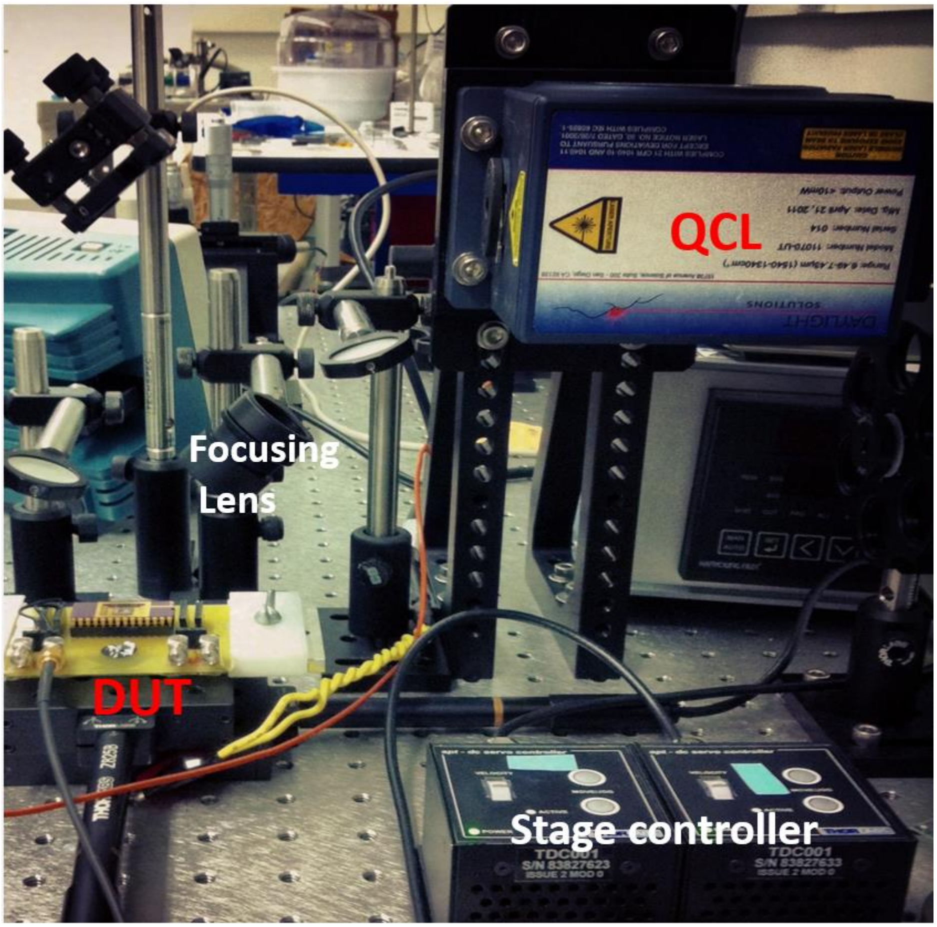

Supplementary Figure 10: Experimental setup for the piezotransistive microcantilever based photoacoustic spectroscopy of analytes. The analytes were deposited near the cantilever base (as shown in the insets of Fig. 7 (a) and (b)). The IR spot was focused on the analytes using the focusing lens arrangement. The quantum cascade laser (QCL) was pulsed at the resonance frequency of the microcantilever (43.93 kHz), and the electrical deflection signal from the HFET was measured. The QCL controller controlled the movement of the stage. The HFET was biased at V_{DS} = 0.5 V, V_{GS} = -2.2 V, and a constant I_{DS} = 100 µA. The lock-in amplifier, SMU and QCL power supplies are not shown in the image.

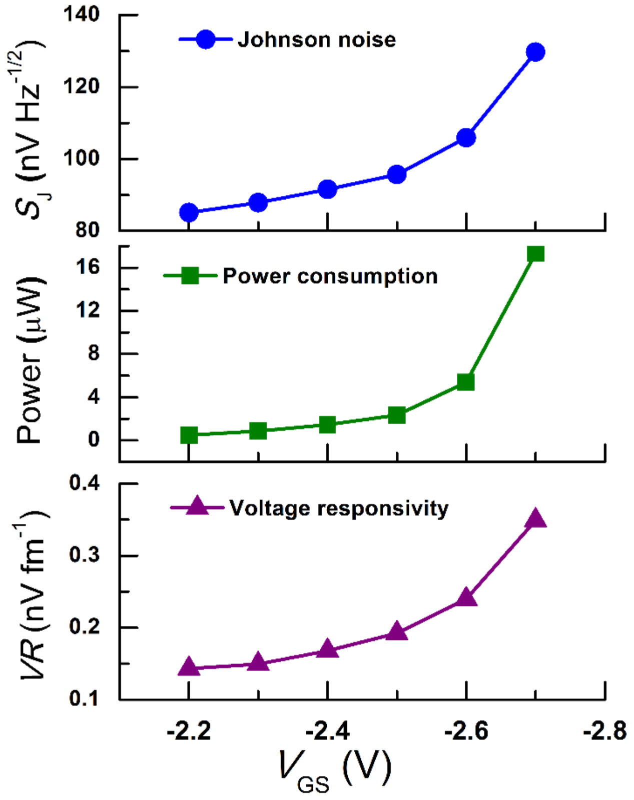

Supplementary Figure 11: Optimization parameters for biasing HFET for dynamic deflection sensing. Three major criteria: electrical noise/Johnson noise (top graph), power consumption (middle graph), and voltage responsivity, *VR* (bottom graph) are shown for different gate bias (V_{GS} = - 2.2 V to -2.7 V) and a constant current, I_{DS} = 10 μA.

Supplementary Table

Supplementary Table 1: Summary of step and dynamic bending performances of Microcantilever 1 (device 1) and microcantilever 2 (device 2)

Device #			1	2
Step bending Performance	Gauge Factor		8700	7300
	Power consumption (nW)		1.36	0.82
Dynamic bending Performance	Resonant Frequency (kHz)		43.934	46.4
	Quality Factor		230	350
	For cantilever oscillation	MDD On resonance (pm $HZ^{-1/2}$)	2.84	3.42
		MDD Off resonance (fm $HZ^{-1/2}$)	60.14	105.2
		Responsivity (nV fm^{-1})	1.43	1.3
	For surface wave excitation	MDD On resonance (fm $HZ^{-1/2}$)	12.35	9.77
		MDD Off resonance (fm $HZ^{-1/2}$)	0.51	2.3

		**Responsivity (nV fm^{-1})**	**170**	**60**

Supplementary Notes

Supplementary Note 1: Formulations for fractional change in piezoresistivity and source-drain resistance

Total resistance of the HFET (externally measured),

$$R_{\mathrm{DS}} = R_{\mathrm{int}} + 2R_{\mathrm{C}} + 2R_{\mathrm{acc}} \tag{1}$$

R_{int} is the drain-source resistance of the intrinsic transistor. R_{c} denotes the source and drain contact resistances (assumed to be equal). R_{acc} is the access region resistance (resistance of the channel from the gate to the source or to the drain, which are also assumed to be equal) and is given by [1],

$$R_{\mathrm{acc}} = \frac{L_{\mathrm{DG}}}{q\mu_{\mathrm{acc}} n_{\mathrm{s,acc}} W_{\mathrm{D}}} = \rho_{\mathrm{acc}} \frac{L_{\mathrm{DG}}}{W_{\mathrm{D}}} \tag{2}$$

Here, L_{DG} is the length of the access region on the drain side and W_{D} is the width of the channel. ρ_{acc}, μ_{acc} and n_{acc} are the sheet resistivity, mobility and 2D sheet carrier concentrations for the

access regions. R_{int} is the drain-source resistance of the intrinsic device, i.e. the resistance of the channel under the gate, and given as [1],

$$R_{\text{int}} = \frac{L_{\text{G}}}{q\mu_{\text{int}} n_{\text{s,int}} W_{\text{D}}} = \rho_{\text{int}} \frac{L_{\text{DG}}}{W_{\text{D}}} \tag{3}$$

Here, ρ_{int}, μ_{int} and n_{int} are the sheet resistivity, mobility and carrier concentrations for the intrinsic device, which can differ significantly from μ_{acc} and n_{acc}, especially with applied gate bias.

Taking differential of both sides of eqn. (1) we get

$$\Delta R_{\text{DS}} = \Delta R_{\text{int}} + 2\Delta R_{\text{acc}} \tag{4}$$

Now, taking differential of both sides of eqn. 3, we have

$$\begin{aligned} \Delta R_{\text{int}} &= \Delta \rho_{\text{int}} \frac{L_{\text{DG}}}{W_{\text{D}}} + \rho_{\text{int}} \Delta \left(\frac{L_{\text{DG}}}{W_{\text{D}}} \right) \\ &= \Delta \rho_{\text{int}} \frac{L_{\text{DG}}}{W_{\text{D}}} + \rho_{\text{int}} \left(\frac{L_{\text{DG}} \Delta W_{\text{D}} + \Delta L_{\text{DG}} W_{\text{D}}}{W_{\text{D}}^2} \right) \end{aligned} \tag{5}$$

Dividing both sides with $R_{int} = \rho_{int}(L_{DG}/W_D)$ we get,

$$\Delta R_{\text{int}} / R_{\text{int}} = \Delta \rho_{\text{int}} / \rho_{\text{int}} + (1 + \upsilon)\varepsilon, \tag{6}$$

where, υ (= $\Delta L_{DG}/\Delta W_D$) is the Poisson's ratio, and ε (= $\Delta W_D/W_D$) is the strain.

Similarly, $\Delta R_{\text{acc}}/R_{\text{acc}} = \Delta\rho_{\text{acc}}/\rho_{\text{acc}} + (1+\upsilon)\varepsilon$ (7)

Dividing both sides of the eqn. 4 with R_{DS} and rearranging, we get,

$$\frac{\Delta R_{\text{DS}}}{R_{\text{DS}}} = \frac{R_{\text{int}}}{R_{\text{DS}}}\left(\frac{\Delta R_{\text{int}}}{R_{\text{int}}}\right) + \frac{R_{\text{acc}}}{R_{\text{DS}}}\left(\frac{2\Delta R_{\text{acc}}}{R_{\text{acc}}}\right) \tag{8}$$

Near the pinch-off region (higher negative V_{GS}), $R_{\text{DS}} \approx R_{\text{int}}$, and $R_{\text{DS}} >> R_{\text{acc}}$. Thus using eqn. 6, eqn. 8 can be written as

$$\frac{\Delta R_{\text{DS}}}{R_{\text{DS}}} \approx \left(\frac{\Delta R_{\text{int}}}{R_{\text{int}}}\right) = \Delta\rho_{\text{int}}/\rho_{\text{int}} + (1+\upsilon)\varepsilon \tag{9}$$

Since, $\rho_{\text{int}} = \frac{1}{q\mu_{\text{int}}n_{\text{s,int}}}$, taking differentials and dividing both sides by ρ_{int} we have the fractional change in piezoresistivity ($\Delta\rho_{\text{int}}/\rho_{\text{int}}$) given as

$$\frac{\Delta\rho_{\text{int}}}{\rho_{\text{int}}} = -\left[{}^{\Delta\mu_{\text{int}}}/_{\mu_{\text{int}}} + {}^{\Delta n_{\text{s,int}}}/_{n_{\text{s,int}}}\right] \tag{10}$$

From eqns. 9 and 10 we get,

$$\frac{\Delta R_{\text{DS}}}{R_{\text{DS}}} \approx -\left[{}^{\Delta\mu_{\text{int}}}/_{\mu_{\text{int}}} + {}^{\Delta n_{\text{s,int}}}/_{n_{\text{s,int}}}\right] + (1+\upsilon)\varepsilon \tag{11}$$

For gate bias near channel pinch-off, $\varepsilon << \Delta n_{\text{s,int}}/n_{\text{s,int}}$ and $\Delta\mu_{\text{int}}/\mu_{\text{int}}$, so we can write

$$\frac{\Delta R_{\mathrm{DS}}}{R_{\mathrm{DS}}} \approx \frac{\Delta \rho_{\mathrm{int}}}{\rho_{\mathrm{int}}} = -\left[\Delta\mu_{\mathrm{int}}/\mu_{\mathrm{int}} + \Delta n_{\mathrm{s,int}}/n_{\mathrm{s,int}}\right] \tag{12}$$

Supplementary Note 2: Optimization of HFET Biasing

In this article, AlGaN/GaN HFETs were used to transduce both the static and dynamic deflections of the microcantilevers. The HFETs were biased considering sensitivity, power consumption, and electrical noise (Johnson noise). Also, the measurement mechanisms are different in the step and dynamic bending operations as described in the main article. From the static bending experiments, it is straight forward that the HFET should be biased as close to the pinch off region which will offer high sensitivity (or *GF*) as can be seen in the inset of Fig. 3 (a). It is also evident from Eqns. (1) and (2) that as gate bias, V_{GS} approaches to channel pinch off, *GF* increases significantly. However for the dynamic deflections, as the deflections are very small, HFET biasing should be optimized based on sensitivity (i.e. voltage responsivity, *VR*), Johnson noise, and the power consumptions. There has to be tradeoffs among these three parameters. Fig. S11 shows the relations of these three parameters with gate bias for device 1. The bottom graph shows that *VR* increases as V_{GS} becomes more negative (closer to pinch off), which supports our step bending scenario. The *VR* was measured as described in the main text and also in the next Supplementary Note 3. However as V_{GS} becomes more negative, R_{DS}

increases more, which increases the power consumed by the HFET as can be seen in the middle graph. The power consumption was calculated using, $P_{\mathrm{DC}} = I_{\mathrm{DS}}^{2} \times R_{\mathrm{DS}}$, where I_{DS} = 10 μA is the constant current supplied from SMU, and R_{DS} was measured from the I-V characteristics of device 1 [see Fig. 2 (a)]. At the same time the Johnson noise ($S_{\mathrm{J}} = \sqrt{4k_{\mathrm{B}}TR_{\mathrm{DS}}\Delta f}$) will also increase with more negative V_{GS}, since R_{DS} increases, as can be seen in the top graph of Fig. S11 (the Johnson noise was measured as described in the main article and also in Supplementary Note 3). Thus, it is clear that the choice should be made according to the applications. As we wanted to detect very small (sub nanometer) displacement, the noise had to be small and the *VR* needed to be high, which means the SNR (signal to noise ratio) should be high. As we are essentially measuring ΔV_{DS} from the HFET (for different deflections), we can significantly increase the voltage signal without affecting the noise by increasing the constant current supply as $\Delta V_{\mathrm{DS}} = I_{\mathrm{DS}} \times \Delta R_{\mathrm{DS}}$, although it sharply increases the power dissipation. Another limitation setting the upper limit of I_{DS} for a given V_{GS} is the saturation current ($I_{\mathrm{DS,sat}}$) since at that current the HFET crosses over from linear region to the saturation region, which can cause V_{DS} and hence power dissipation to increase uncontrollably and damage the device. Of course, the current source should also be able to reliably supply high constant current at the resonant frequency, which can also be a limitation. Considering all these factors for the detection of femtoscale displacement, we thus optimized the biasing conditions as: V_{GS} = - 2. 2 V, I_{DS} = 100 μA, which offered *VR* = 1.43 nV fm^{-1} for $S_{\mathrm{J}} \approx$ 86 nV Hz$^{-1/2}$ and power consumption of 51 μW. From Fig. S11, the *VR* for this biasing scheme is much higher and the noise is much lower compare to V_{GS} = - 2. 7 V, I_{DS} = 10 μA biasing condition, with the increase in power consumption. For ultra-high frequency operations (> 1 MHz), readers are advised to refer to [2-4].

Supplementary Note 3: Calculations of noises, responsivity, and minimum detectable displacement (MDD)

The Johnson noise and thermo-mechanical noise for a cantilever are given by

Johnson noise (V): $S_{\mathrm{E}}^{1/2} = \sqrt{4k_{\mathrm{B}}TR_{\mathrm{DS}}\Delta f}$ (1)

Thermo-mechanical noise at resonance: $S_{\mathrm{TM,on}}^{1/2} = \sqrt{4k_{\mathrm{B}}TQ\Delta f/(2\pi f_0 K)}$ (2)

Thermo-mechanical noise off resonance (nm): $S_{\mathrm{TM,off}}^{1/2} = \sqrt{4k_{\mathrm{B}}TQ\Delta f/(2\pi f_0 QK)}$ (3)

The two important noise sources for microcantilevers with electrical readouts are Johnson noise and Thermomechanical noise, which we calculated following the same procedure as discussed in Ref. 2. For V_{GS} = - 2.2 V and V_{DS} = 0.5 V, R_{DS} was found from Fig. 2 (a) to be 5 kΩ and the Johnson noise using (1) , was calculated as 9.12 nV Hz$^{-1/2}$ for a measurement bandwidth (Δf) of 1 Hz, and using $k_{\mathrm{B}}T$ = 0.026 eV at room temperature. However the voltage noise spectral density (in the inset of Fig. 4 (b)) was 86 nV Hz$^{-1/2}$ which actually incorporates other noise sources, such as, current preamplifier, dynamic signal analyzer, cables, etc., in addition to the Johnson noise. On the other hand, for the quality factor, Q = 230, resonant frequency, f_0 = 43.934 kHz (from the electrical readout as shown in Fig. 4 (a)), spring constant, K = 1.71 N/m (estimated from COMSOL), and measurement bandwidth, Δf = 1 Hz, the thermomechanical noise (using (2)) was calculated as 2.84 pm Hz$^{-1/2}$ (on resonance) and 12.38 fm Hz$^{-1/2}$ (using (3) for off resonance). From Fig. 4 (b), the measured voltage noise spectral density on and off resonance are 4.07 μV Hz$^{-1/2}$ and 86 nV Hz$^{-1/2}$, respectively. Thus the contribution related to the cantilever's thermomechanical motion was estimated as 4.07 μV Hz$^{-1/2}$ $\left(= \sqrt{(4.07\ \mu\mathrm{V})^2 - (86\ \mathrm{nV})^2}\ \mathrm{Hz}^{-\frac{1}{2}}\right)$.

As a result the transduction gain or displacement responsivity was calculated as 1.43 nV fm^{-1} (4.07 μV $Hz^{-1/2}$/2.84 pm $Hz^{-1/2}$). Moreover the off-resonance noise limited displacement resolution (or minimum detectable displacement (MDD)) was estimated as 60.14 fm $Hz^{-1/2}$ (86 nV $Hz^{-1/2}$/1.43 nV fm^{-1}). Using the responsivity value of 1.43 nV fm^{-1}, the calculated voltage was 12.44 μV for the oscillation amplitude of 8.7 pm which excellently matches with the experimental observations as shown in Fig. 4(a). Following similar approach, for device 2, the measured noise limited displacement resolution was found to be 3.42 pm $Hz^{-1/2}$ (on resonance) and 105.2 fm $Hz^{-1/2}$ (off resonance including measurement noise) with a displacement responsivity of 1.3 nV fm^{-1}.

Supplementary References

[1] Dang, X. Z. *et al*. Measurement of drift mobility in AlGaN/GaN heterostructure field-effect transistor. *Appl. Phys. Lett.* **74**, 3890-3892 (1999).

[2] Faucher, M. *et al*. Amplified piezoelectric transduction of nanoscale motion in gallium nitride electromechanical resonators. *Appl. Phys. Lett*. **94**, 233506 (2009).

[3] Ansari, A. & Rais-Zadeh, M. R. A Thickness-Mode AlGaN/GaN Resonant Body High Electron Mobility Transistor. *IEEE Trans. Electron Devices* **61**, 1006-1013 (2014).

[4] Rais-Zadeh, M. *et al*. Gallium Nitride as an Electromechanical Material. *J. Microelectromech. Syst*. **23**, 1252-1271 (2014).
